# Development of a Cloud-Based Learning Module for Protein Crystallography

**DOI:** 10.1063/4.0000818

**Published:** 2025-10-27

**Authors:** Christopher Jurgenson

**Affiliations:** Delta State University, Cleveland, MS, USA

## Abstract

The National Institute of General Medical Sciences (NIGMS) Sandbox, in collaboration with the NIH Office of Data Science Strategy (ODSS), NIH Center for Information Technology (CIT), and Deloitte, has developed an innovative, cloud-based training module on protein crystallography. This module, designed to enhance accessibility and scalability, is hosted on Amazon Web Services (AWS) and is part of a broader initiative to provide free, interactive training in biomedical data science. The protein crystallography module is divided into three submodules, each focusing on a critical aspect of the crystallographic process: (1) an introduction to protein structure and crystallographic theory, (2) solving structures using single-wavelength anomalous diffraction (SAD) phasing, and (3) model building and refinement using molecular replacement.

The first submodule provides a foundational understanding of protein structure, emphasizing the relationship between structure and function, and introduces key concepts in crystallography, including crystallization strategies and the phase problem. The second submodule delves into SAD phasing, guiding users through the process of solving protein structures using anomalous diffraction data. The third submodule focuses on model building and refinement, utilizing the Crystallographic Object-Oriented Toolkit (Coot) and the Phenix software suite to teach students how to interpret electron density maps, build protein models, and refine structures for deposition into the Protein Data Bank (PDB).

Each submodule integrates Jupyter Notebooks, GitHub repositories, and cloud computing resources to provide a hands-on, interactive learning experience. The module also includes quizzes and practical exercises to reinforce key concepts, such as selecting search molecules for molecular replacement, using Coot to build protein structures, and refining models with Phenix. By leveraging the NIGMS Sandbox’s cloud-based infrastructure, this module enables researchers and students from diverse institutions to gain practical experience with protein crystallography software and concepts, thereby accelerating their ability to apply these techniques in their own research.

